# Krüppel-like factor 6 regulates transforming growth factor-β gene expression during human respiratory syncytial virus infection

**DOI:** 10.1186/1743-422X-8-409

**Published:** 2011-08-17

**Authors:** Victoria Mgbemena, Jesus Segovia, TeHung Chang, Santanu Bose

**Affiliations:** 1 Department of Microbiology and Immunology, The University of Texas Health Science Center at San Antonio, San Antonio, Texas, USA

**Keywords:** Krüppel-like factor 6, human respiratory syncytial virus, transforming growth factor-β, gene expression, transcription factor

## Abstract

**Background:**

Human respiratory syncytial virus (RSV) infection is associated with airway remodeling and subsequent asthma development. Transforming growth factor-beta (TGF) plays a crucial role in asthma development. The mechanism regulating TGF gene expression during RSV infection is not known. Kruppel-like factor family of transcription factors are critical regulators of cellular/tissue homeostasis. Previous studies have shown that Kruppel-like factor 6 (KLF6) could function as a trans-activator of TGF gene; however, whether KLF members play a role during infection is unknown. In the current study we have evaluated the role of KLF6 during TGF expression in RSV infected cells.

**Findings:**

Silencing KLF6 expression by shRNA led to drastic inhibition in TGF production during RSV infection, as assessed by ELISA analysis of medium supernatants. RT-PCR analysis revealed loss of TGF expression in KLF6 silenced cells. Chromatin-immunoprecipitation assay conducted with RSV infected cells showed binding of KLF6 protein to the TGF promoter during RSV infection. We further observed reduced RSV infectivity in KLF6 silenced cells and in cells incubated with TGF neutralizing antibody. In contrast, enhanced RSV infection was noted in cells incubated with purified TGF.

**Conclusion:**

We have identified KLF6 as a key transcription factor required for trans-activation of TGF gene during RSV infection. Moreover, TGF production is required for efficient RSV infection and thus, KLF6 is also required for efficient RSV infection by virtue of KLF6 dependent TGF production during infection.

## Findings

Human respiratory syncytial virus (RSV) is a non-segmented negative strand single-stranded RNA (NNS) virus that causes severe lung diseases upon infection of airway epithelial cells. RSV infection among high risk individuals (e.g. infants, children, immuno-compromised individuals) manifests in inflammatory diseases like bronchiolitis and pneumonia [1]. It is also evident that airway remodeling during RSV infection leads to asthma development and exacerbation [2,3].

One of the hallmarks of RSV infection is enhanced airway hyper-responsiveness due to airway remodeling. Airway remodeling leads to asthma development and RSV infection has been linked with progression and exacerbations of asthma [2,3]. Transforming growth factor-β (TGF-β) production during RSV infection may play a role in asthma development, since TGF-β is a key player associated with asthma development [4-7]. TGF-β also regulates immune response against RSV infection of infants by modulating cytokine production [8]. Although TGF-β plays an important role during RSV-induced lung disease infection, the mechanism regulating TGF-β gene expression during RSV infection is unknown. In the current study, we have identified Krüppel-like factor 6 **(**KLF6) as a critical transcription factor required for TGF-β gene-expression during RSV infection of human lung epithelial cells. Although Krüppel-like factor (KLF) transcription factor family regulates important biological processes [9], their role during infection was not known. Herein, we have uncovered the ability of Krüppel-like factors like KLF6 to function as a trans-activator of a host gene (i.e. TGF-β gene) during virus (RSV) infection.

### KLF6 positively regulates TGF-β gene expression

**Figure 1:**
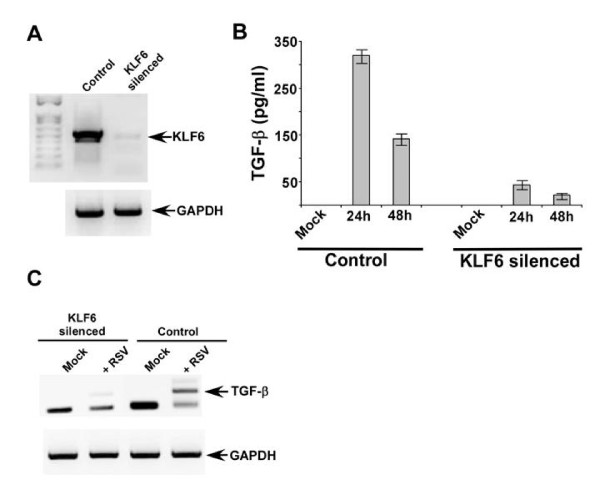
**KLF6 is required for TGF-β gene expression**. **(a) **RT-PCR analysis of KLF6 expression in stable A549 cells expressing either scrambled shRNA (control) or KLF6-specific shRNA (KLF6 silenced cells). A549 cells stably expressing KLF6 shRNA was generated by tranducing with lentivirus expressing KLF6 shRNA. **(b) **TGF-β production from mock and RSV infected control and KLF6 silenced cells. TGF-β was measured by ELISA and each value represents the mean ± standard deviation from three independent experiments. **(c) **RT-PCR analysis of TGF-β expression in control and KLF6 silenced cells infected with RSV for 24 h. The gels shown in (a) and (c) are representative of three independent experiments that yielded similar results.

A549 cells are routinely used as model type-II human alveolar epithelial cells and the alveolar cells are specifically infected by RSV during productive infection of human airway. A stable cell line lacking KLF6 was generated from A549 cells by utilizing KLF6-specific shRNA expressing lentiviral particles (Santa Cruz Biotechnology, CA, USA). The efficiency of silencing is evident from lack of KLF6 mRNAs in cells stably expressing KLF6 specific shRNA (Figure 1). KLF6 mRNA was assessed by reverse transcription-PCR or RT-PCR described previously [10,11]. The control cells represent stable cells that were generated following transduction of lentivirus expressing scrambled shRNA (Santa Cruz Biotechnology, CA, USA). The primers utilized for the RT-PCR assay is listed in table-1.

**Table 1:** RT-PCR primers

Gene name	Forward	reverse
Human GAPDH	5'-GTCAGTGGTGGACCTGACCT	5'-AGGGGTCTACATGGCAACTG

Human KLF6	5'CTCTCAGCCTGGAAGCTTTTAGCCTAC	5'-ACAGCTCCGAGGAACTTTCTCCCA

Human TGF-β	5'-CGCGTGCTAATGGTGGAAA	5'-CGCTTCTCGGAGCTCTGATG.

Control and KLF6 silenced cells were infected with purified RSV (1 MOI) [10-13], since KLF6 regulates TGF-β gene expression during hepatic fibrosis [14,15] and thus, we examined whether KLF6 play a similar role during virus infection. Medium supernatant derived from control and KLF6 silenced cells infected with RSV were used to detect TGF-β protein levels by TGF-β ELISA kit from eBioscience, CA, USA. Total RNA collected from these cells was used to examine TGF-β mRNA levels by RT-PCR. TGF-β expression/production is regulated by KLF6; since drastic reduction in TGF-β production was observed from infected cells devoid of KLF6 (Figure 1B). The loss of secreted TGF-β protein was due to reduced TGF-β mRNA levels in KLF6 silenced cells (Figure 1C). Thus, our results demonstrated that KLF6 positively regulates TGF-β transcription during RSV infection of lung epithelial cells.

### Binding of KLF6 to TGF-β promoter during RSV infection

Chromatin immuno-precipitation (ChiP) assay [16] was performed to examine binding of KLF6 to TGF-β promoter during RSV infection. Chromatin isolated from RSV (1 MOI) infected A549 cells was immuno-precipitated overnight at 4°C with either anti-KLF6 antibody or isotype matched control IgG (Santa Cruz Biotechnology, CA, USA). The precipitated DNA was analyzed using promoter-specific PCR primer pairs encompassing the KLF6 binding site at the TGF-β promoter. PCR primer pairs corresponding to the TGF-β promoter site that do not bind to KLF6 served as a negative control. The KLF6-DNA complex was analyzed by standard PCR using primer specific for KLF6 binding site on TGF-β promoter [14]. The primer sequences used for the ChiP assay are listed in table-2.

**Table 2:** ChiP primers

Encompassing the KLF6 binding site at the TGF-β promoter	TGF-β forward 5' -AAGGAGGCTGGGTTGGAAACTC-3' TGF-β reverse ' 5 -TGGGACCACACCTGGAAATG-3'	Product: 181 bp (encompassing -85 to -265 region of TGF-β promoter)	Optimal annealing temp: 58°C.
Encompassing the region in TGF-β promoter that does not bind to KLF6 (negative control)	TGF-β forward 5' -CCTCTTTCTCTGGTGACCCAC-3' TGF-β reverse ' 5 -CCACCGTCCTCATCTCGC-3'	Product: 185 bp (encompassing -1028 to -1212 region of TGF-β promoter)	Optimal annealing temp: 60.6°C.

**Figure 2:**
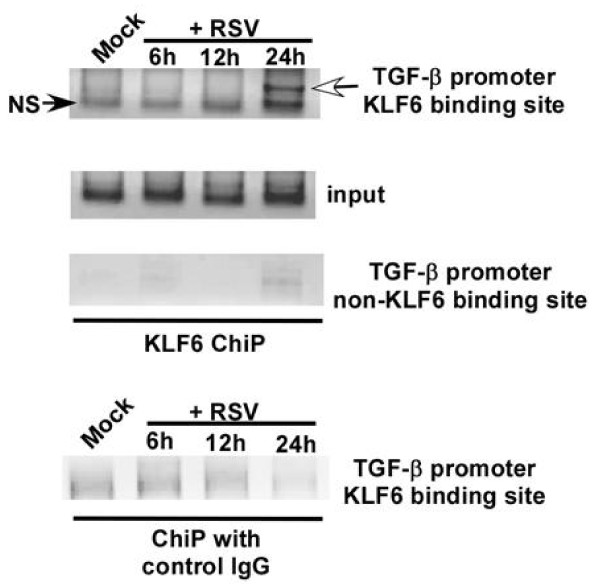
**Binding of KLF6 to TGF-β promoter during RSV infection**. ChiP assay on A549 cells that were either mock infected or infected with RSV for 6 h, 12 h and 24 h. Both anti-KLF6 antibody and isotype matched control antibody was used. An upstream region in the TGF-β gene devoid of the KLF6 responsive region was probed as a negative control. NS; non-specific. The white arrowhead denotes KLF6 binding to the TGF-β promoter. The ChiP assay gels are representative of three independent experiments that yielded similar results.

KLF6 binding to TGF-β promoter was not observed in un-infected cells, whereas RSV infection resulted in binding of KLF6 to TGF-β promoter at 24 h post-infection (Figure 2), which corresponds to maximal TGF-β production from infected cells (Figure 1B). KLF6 binding to TGF-β promoter was specific, since immuno-precipitation with control IgG did not yield any amplified product. Similarly, no amplified product was observed following amplification with primers that do not correspond to KLF6 binding site on TGF-β promoter. These studies demonstrated that KLF6 directly trans-activates TGF-β gene expression following its association with TGF-β promoter during RSV infection.

### RSV replication is regulated by KLF6

**Figure 3:**
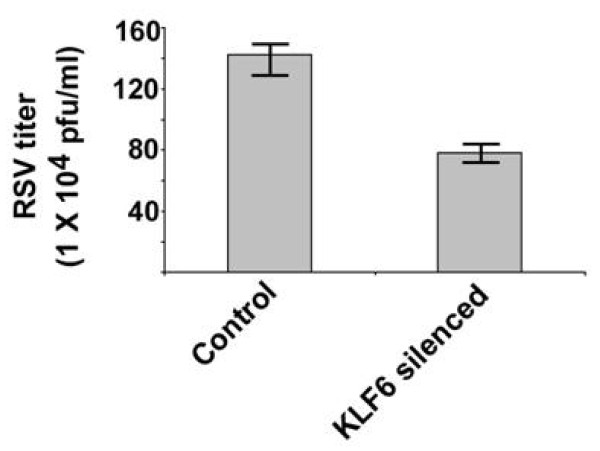
**KLF6 regulates RSV infection**. RSV titer (measured by plaque assay of medium supernatant) at 24 h post-infection of control and KLF6 silenced cells. The plaque assay values represent the mean ± standard deviation from three independent experiments.

Since KLF6 is critical for expression of TGF-β, a gene that is known to modulate RSV infection [6,7], we next evaluated the role of KLF6 in regulating RSV infectivity. For these studies, control and KLF6 lacking cells were infected with RSV. Medium supernatant collected from RSV infected control and KLF6 lacking cells were used to determine infectious viral titer by plaque assay. Expression of KLF6 is also required for RSV replication, since number of infectious plaque (representing viral infectivity/replication) was reduced by approximately 40% in KLF6 silenced cells (Figure 3). This result implicated an essential role of KLF6 in supporting optimal RSV replication/infection in lung epithelial cells.

### KLF6 mediated production of TGF-β is required for optimal RSV infectivity

**Figure 4:**
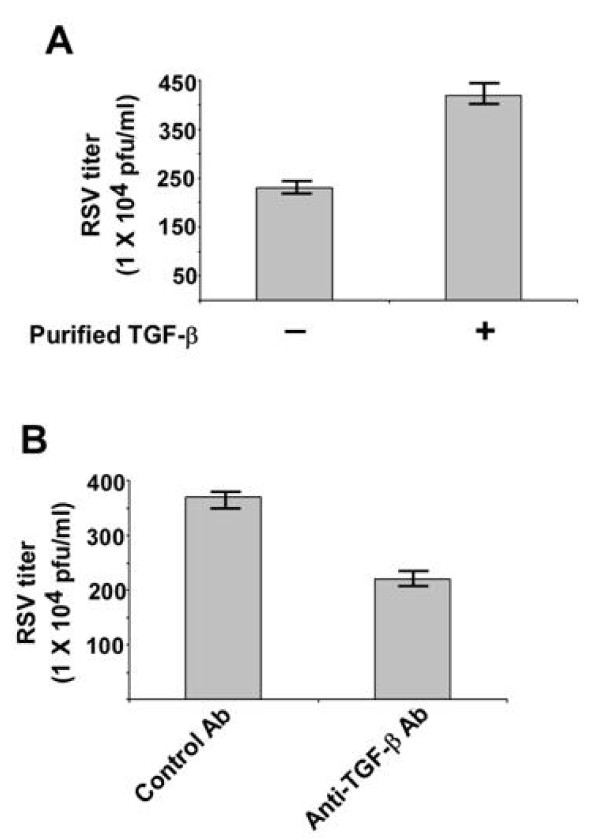
**TGF-β controls RSV infection**. **(a) **RSV titer at 24 h post-infection of A549 cells that were infected in the absence or presence of purified TGF-β. **(b) **RSV titer at 24 h post-infection of A549 cells that were infected in the presence of either control antibody (Ab) or TGF-β neutralizing antibody. For the above experiments, A549 cells were pre-treated with antibodies or purified TGF-β for 2 h prior to RSV infection and it was present during infection. The plaque assay values represent the mean ± standard deviation from three independent experiments.

Since KLF6 expression is crucial for optimal RSV infectivity (Figure 3), we next investigated whether TGF-β confers such activity. A549 cells were pre-incubated with purified TGF-β (a kind gift from Dr. LuZhe Sun, University of Texas Health Science Center) for 2 h prior to RSV infection. RSV infection was performed either in the presence or absence of TGF-β protein. Viral infectivity was assessed by plaque assay of medium supernatant. As shown in Figure 4A, treatment of cells with purified TGF-β enhanced viral infectivity by approximately 160%, suggesting that indeed TGF-β posses pro-RSV activity.

The role of TGF-β during RSV infection was further confirmed by using neutralizing anti-TGF-β antibody (anti-TGF). A549 cells pre-incubated (for 2 h) with either isotype matched control antibody (control IgA2) or anti-TGF-β (anti-human TGF-β IgA) were infected with RSV in the presence or absence of the corresponding antibodies. Control and TGF-β neutralizing antibodies were purchased from Invivogen, San Diego, CA. Viral infectivity was assessed by plaque assay analysis of medium supernatant. Neutralization of TGF-β activity during RSV infection resulted in reduction of viral titer by approximately 40% (Figure 4B). Interestingly, the reduction in viral titer was similar to that was observed following infection of KLF6 silenced cells (Figure 3). These results demonstrated that KLF6 mediated production of TGF-β is required for optimal RSV infectivity.

TGF-β gene expression is tightly regulated since TGF-β contributes to development of chronic disorders like asthma [4,5]. Infection with respiratory viruses like RSV and rhinovirus leads to development of asthma, probably due to airway remodeling by TGF-β produced during infection [4,5,17]. TGF-β also regulates RSV infectivity and immune response in infected host. For example, previous studies have reported the requirement of TGF-β to support RSV replication [6,7] and regulate the immune response in infected infants [8]. Despite the multi-functional role of TGF-β during RSV infection, the mechanism regulating TGF-β gene expression during RSV infection was unknown. In the current study, we have identified KLF6 as a critical transcription factor required for TGF-β gene-expression/production during RSV infection. Furthermore, we demonstrated that both KLF6 and TGF-β is required for optimal RSV infection of lung epithelial cells.

The family of Krüppel-like factor (KLF) transcription factors controls a wide spectrum of biological processes including cell growth, cell proliferation and differentiation [9]. KLF transcription factors regulate the function of various organ systems (hematological, digestive, cardiovascular, respiratory systems) and have been implicated in the development/progression of diseases like cancer, metabolic disorders, cardiovascular and inflammatory diseases [9]. KLF6 factor is known to regulate gene expression in various tissues by acting as a trans-activator or repressor of gene expression [18,19]. In addition, KLF6 has been identified as a tumor suppressor gene associated mainly with prostate cancer [20]. KLF6 mediated TGF-β expression was shown to contribute to the development of hepatic fibrosis [15,21]. During hepatic fibrosis, KLF6 directly associated with the TGF-β gene [14,15]. Although KLF factors have been implicated in regulating normal cellular/tissue homeostasis, their role during infection has not been examined yet. In the present study we have shown that indeed, KLF factors like KLF6 play an essential role during infection by regulating the expression of TGF-β gene during RSV infection.

Our studies have demonstrated that KLF6 directly binds to TGF-β promoter during RSV infection and this event results in trans-activation of TGF-β gene. Interestingly, KLF6 is expressed in the lungs [22] and KLF4 (both KLF4 and KLF6 are phylogenitically related since both belong to group-2 of KLF factors) expression in lung is potentially associated with pulmonary fibrosis and enhanced inflammation of the airway [23]. Thus, we speculate that similar to KLF4, KLF6 may play a similar role during RSV infection of the respiratory tract since KLF6 regulates expression of TGF-β, which is involved in lung remodeling and airway fibrosis. Further studies are required to establish these regulatory functions of KLF6 by using conditionally knockout transgenic mouse once they are available because KLF6 knockout results in embryonic lethality.

## Abbreviations

RSV: human respiratory syncytial virus; TGF-β: transforming growth factor-β; KLF6: Krüppel-like factor 6; ChiP: chromatin immunoprecipitation

## Competing interests

The authors declare that they have no competing interests.

## Authors' contributions

VM, JS and SB designed the experiments and prepared the manuscript. VM, JS and TC performed the experiments. All authors read and approved the final manuscript.
